# Candidate innate immune system gene expression in the ecological model *Daphnia*

**DOI:** 10.1016/j.dci.2011.04.004

**Published:** 2011-10

**Authors:** Ellen Decaestecker, Pierrick Labbé, Kirsten Ellegaard, Judith E. Allen, Tom J. Little

**Affiliations:** a Aquatic Biology, Science & Technology, IRF-Life Sciences, K.U.Leuven-Campus Kortrijk, Belgium; b Aquatic Ecology and Evolutionary Biology, K.U.Leuven, Belgium; c University of Edinburgh, IEB, Edinburgh, UK; d ISEM, University of Montpellier 2, France; e Department of Molecular Evolution, Evolutionary Biology Centre, Uppsala University, Sweden

**Keywords:** Host–pathogen coevolution, qRT-PCR, *Pasteuria*, Parasitism, Resistance, Innate immune system

## Abstract

The last ten years have witnessed increasing interest in host–pathogen interactions involving invertebrate hosts. The invertebrate innate immune system is now relatively well characterised, but in a limited range of genetic model organisms and under a limited number of conditions. Immune systems have been little studied under real-world scenarios of environmental variation and parasitism. Thus, we have investigated expression of candidate innate immune system genes in the water flea *Daphnia*, a model organism for ecological genetics, and whose capacity for clonal reproduction facilitates an exceptionally rigorous control of exposure dose or the study of responses at many time points. A unique characteristic of the particular *Daphnia* clones and pathogen strain combinations used presently is that they have been shown to be involved in specific host–pathogen coevolutionary interactions in the wild. We choose five genes, which are strong candidates to be involved in *Daphnia*–pathogen interactions, given that they have been shown to code for immune effectors in related organisms. Differential expression of these genes was quantified by qRT-PCR following exposure to the bacterial pathogen *Pasteuria ramosa*. Constitutive expression levels differed between host genotypes, and some genes appeared to show correlated expression. However, none of the genes appeared to show a major modification of expression level in response to *Pasteuria* exposure. By applying knowledge from related genetic model organisms (e.g. *Drosophila*) to models for the study of evolutionary ecology and coevolution (i.e. *Daphnia*), the candidate gene approach is temptingly efficient. However, our results show that detection of only weak patterns is likely if one chooses target genes for study based on previously identified genome sequences by comparison to homologues from other related organisms. Future work on the *Daphnia–Pasteuria* system will need to balance a candidate gene approach with more comprehensive approaches to *de novo* identify immune system genes specific to the *Daphnia*–*Pasteuria* interaction.

## 1 Introduction

Parasitic infection is often associated with genetic differences between hosts. Resistant genotypes should increase in frequency, but evolution towards greater immunity in hosts can be matched by counter-adaptation in pathogens, possibly leading to cycles of adaptation and counter adaptation, i.e. coevolution. Unfortunately, despite widespread knowledge of the occurrence of genetic variation for resistance in hosts (and indeed, strain variation in pathogens), we lack understanding of the coevolutionary dynamic. For instance, the coevolutionary process could largely be due to selective sweeps where genetic polymorphism is transient, observable only briefly as one genotype rises to fixation, leaving populations monomorphic until the next mutation arises (Woolhouse et al., 2002). Alternatively, there could be frequency dependent selection where common genotypes are disfavoured, such that no single genotype can go to fixation and rare genotypes are unlikely to ever go extinct (Decaestecker et al., 2007; Schulte et al., 2010). As a further alternative, polymorphic states could be widely maintained by trade-offs where high resistance is costly in the absence of parasitism (Gandon et al., 2008; Gaba and Ebert, 2009; Säppala and Jokela, 2010).

Each of these flavours of parasitic interaction has some support in the literature. Evidence for selective sweeps is present in the coevolution between bacteria and their phages (Buckling and Rainey, 2002; Brockhurst et al., 2003) and has been widely detected in DNA sequences coding for immune proteins of both *Drosophila* (Schlenke and Begun, 2004) and rodents (Hurst and Smith, 1999). Trade-offs between resistance to biological enemies and fitness in the absence of attack has arisen in laboratory lines selected for resistance (e.g. Kraaijeveld and Godfray, 1997), though other types of study design have not always detected trade-offs, including two on *Daphnia* (Little et al., 2002; Altermatt and Ebert, 2007). The potential for frequency dependent selection due to pathogens has been indicated by experimental studies showing that the probability of infection depends on both the genetic background of hosts, and the genetic background of the infecting pathogens, a pattern termed genetic specificity (Carius et al., 2001). Frequency-dependent selection is extensively documented in the snail *Potamopyrgus antipodarum* during trematode infections. This has been achieved via experimental manipulations under semi-natural conditions, as well as through field collections where recently common clones often are the most heavily infected, and tend to decline in frequency (Koskella and Lively, 2009; Jokela et al., 2009). This frequency-dependent field pattern required intensive and relatively long-term longitudinal sampling. A unique alternative is the resurrection of host and pathogens in diapause from a layered seed bank. This was achieved with the crustacean *Daphnia* where carefully controlled hatching from different layers allowed host and pathogens from different time points to be compared in a ‘time-shift’ experiment, reconstructing host–parasite coevolution from the past (Decaestecker et al., 2007). In sum, however, there are not enough longitudinal or time-shift studies completed to conclude that frequency dependent is to commonest mode of coevolution.

Tracking genetic change over time can be greatly augmented by identifying key genes of the infection process, and in particular genes whose sequence polymorphisms are linked to variation in susceptibility. This should allow the high-throughput tracking of genotype frequencies. Opportunely, the last ten years have witnessed a renewed interest in the invertebrate innate immune system (Kurtz and Armitage, 2006; Schulenburg et al., 2009), and it is now relatively well understood in some invertebrate genetic model organisms, particularly in *Drosophila melanogaster* (Hoffmann, 2003; Brennan and Anderson, 2004) and *Anopheles gambiae* (see review in Christophides et al., 2004). Three types of components have been identified: (i) receptors, which recognize pathogen associated molecular patterns (PAMPs), (ii) regulators, which are implied in signalling pathways (e.g. the Toll and Imd pathways) and (iii) effectors, which directly inhibit pathogen growth or survival (Schmid-Hempel, 2005; Sackton et al., 2007). These effector systems are primarily based on phagocytic cells that, in addition to engulfing foreign particles, generate reactive oxygen and nitrogen species that destroy pathogens.

*Daphnia* represents a powerful model system for coevolution and ecology of host–parasite interactions, and one for which a substantial database is available as a reference, thus facilitating placement of immunological data into a natural context (Orsini et al., 2010). Here, we begin the empirical effort to characterise the innate immune response of a host–pathogen system, *Daphnia magna* and its bacterial pathogen *Pasteuria ramosa*. Our longer-term aim is to track coevolutionary dynamics in the wild and in great detail, but characterisation of the host immune response is an important foundation for this aim. From earlier phenotypic work (Decaestecker et al., 2007), we know how infectivity changes as *Daphnia* and pathogens are coevolving, but we do not know which immune system genes are involved. If we can identify such immune system genes, we would have a powerful tool for investigating the mechanisms of invertebrate immunity, immunity across evolutionary time, and links between invertebrate immunity and other traits. With this in mind, we studied the abundance of RNA transcripts of a suite of candidate immune system genes, which are well-established in other invertebrates to be part of the immune effector systems. This work was made possible by the recent sequencing of the genome of *Daphnia pulex*, and the specific characterisation of putative immune system genes (McTaggart et al., 2009). We used the sequences of putative *D*. *pulex* immune system genes to design PCR primers for use with *D*. *magna*, the latter species being the only *Daphnia* species that is used as model for the study of parasitism. Key to our study was the choice of host and pathogen samples. We used (1) a set of host and pathogen genotypes that show strong genetic specificity (Carius et al., 2001), implying that the population from which they were derived harbours considerable potential for frequency-dependent coevolutionary dynamics; (2) a set of hosts that were derived from the same sediment core that was previously part of a time-shift experiment (Decaestecker et al., 2007). The clonal nature of *Daphnia* facilitated accurate partitioning of the effects of exposure and genetic background, as well as the study of expression changes at independent time points.

## 2 Results

In the pathogen exposure treatments, high levels of infection were obtained for the GG4 clone when exposed to the Sp1 strain, but not when exposed to the Sp8 strain (Exp. A–C, and F; Table 1). By contrast, infections were obtained for the GG13 clone after exposure to the Sp8 strain, but not to the Sp1 strain (Exp. B and C; Table 1). Finally, the GG7 clone was never infected (Exp. A–C, and E; Table 1), which was expected based on past work with this genotype. *Pasteuria* exposure of the Belgian *D. magna* clones generated a high level of infection in both *Daphnia* clones (infection rates were 0.91 and 0.61 for 11.3 and 12.2, respectively, Exp. D; Table 1).

**Fig. 1:**
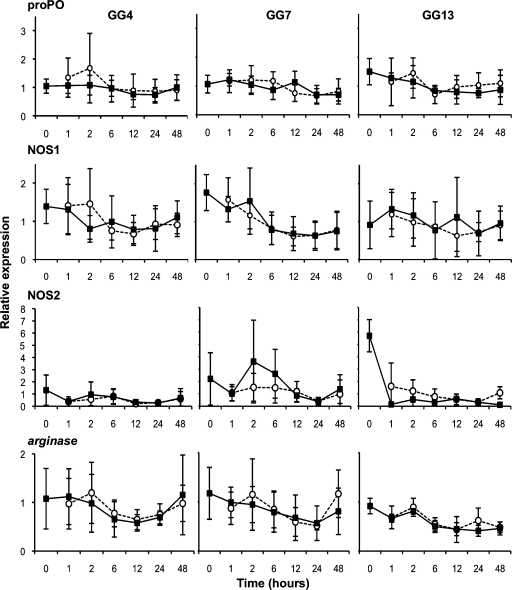
Short-term relative expression of candidate immune system genes in Experiment A&B (Sp1, German clones). The expression of the candidate immune system genes relative to the expression of reference genes of the German *Daphnia* clones exposed to the Sp1 *Pasteuria* strain is given. For each gene and genotype, the mean expression in individuals exposed to the pathogen (white circles and dotted line) or to the placebo solution (black squares and plain line) is presented; error bars represent the standard error. GG13 was included only in Experiment B. Data from the two experiments are combined in the figure, as statistical analyses did not found significant differences between these replications of the same protocol. See statistics in Table 2.

In the short-term (Exp. A–C) and longer term (Exp. E) experiments on the German samples we did not detect any significant interactions of interest. However, for NOS2, we found a significant Geno effect (Table 2), NOS2 being constitutively more expressed in the resistant GG7 *Daphnia* clone than in the GG4 or GG13 *Daphnia* clone (approximately twice more and effect strongest present in Exp. A&B, Fig. 1). In Experiment D (Belgian clones and pathogen strain), we again detected no significant interactions of interest. For NOS1 in the Belgian samples, we found a significant Geno effect (Table 3), indicating that NOS1 is constitutively more expressed in the *Daphnia* clone 11.3 than in clone 12.2 (Fig. 3). In Experiment E, which studied the German clones under longer time frames, we detected a significant Expo effect (Table 2) for the proPO genes, due to relative high expression in the last time point in the GG4 and the GG7 *Daphnia* clone, and again found that NOS2 is constitutively more expressed in the GG7 than in the GG4 *Daphnia* clone (Table 2 and Fig. 4).

**Fig. 2:**
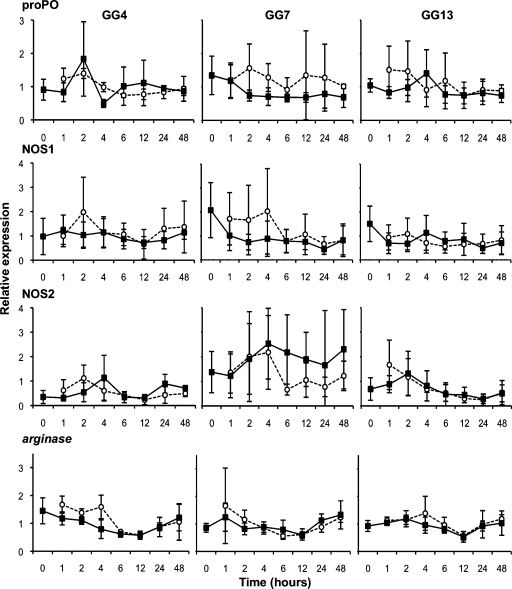
Short-term relative expression of candidate immune system genes in Experiment C (Sp8, German clones). The expression of the candidate immune system genes relative to the expression of reference genes of the German *Daphnia* clones exposed to the Sp8 *Pasteuria* strain is given. For each gene and genotype, the mean expression in individuals exposed to the pathogen (white circles and dotted line) or to the placebo solution (black squares and plain line) is presented; error bars represent the standard error. See statistics in Table 2.

**Fig. 3:**
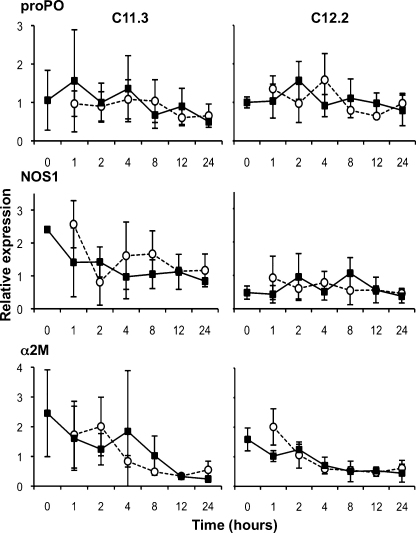
Short-term relative expression of candidate immune system genes in Experiment D (Sp1, Belgian clones). The expression of the candidate immune system genes relative to the expression of reference genes in the Belgian *Daphnia* clones is given. For each gene and genotype, the mean expression in individuals exposed to the pathogen (contemporary pathogen; white circles and dotted line) or to the placebo solution (black squares and plain line) is presented; error bars represent the standard error. See statistics in Table 3.

**Fig. 4:**
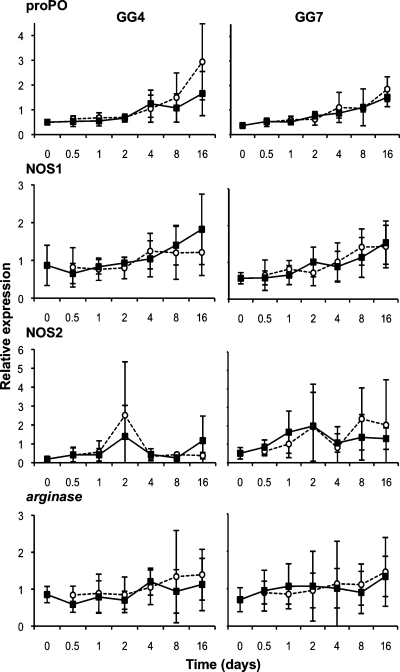
Long-term relative expression of candidate immune system genes in Experiment E (Sp1, German clones). The expression of the candidate immune system genes relative to the expression of the reference genes is given. For each gene and genotype, the mean expression in individuals exposed to the pathogen (Sp1 *Pasteuria* strain; white circles and dotted line) or to the placebo solution (black squares and plain line) is presented; error bars represent the standard error. See statistics in Table 2.

**Fig. 5:**
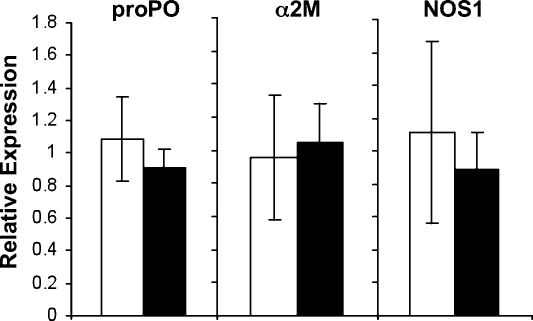
Two-hours post-exposure relative expression of candidate immune system genes in Experiment F (Sp1, German clones). The expression of the German GG4 *Daphnia* clone relative to the expression of reference genes is given. For each gene, the mean expression in individuals exposed to the parasite (Sp1 *Pasteuria* strain; white bars) or to the placebo solution (black bars) is presented; error bars represent the standard error. See statistics in text.

In many of the short-term experiments (Exp. A–D) there seemed to be a trend for expression modification in the early hours after exposure for proPO, NOS1 and α2M (2 h time-point, Figs. 1–3). Experiment F therefore sought to study this time point more intensively. While there was a similar trend in this experiment, it did not show to be significant, i.e. there was no effect of exposure to the pathogen (Expo, Fig. 5; proPO: *F* = 3.01, *P* = 0.1; α2M: *F* = 0.93, *P* = 0.35; NOS1: *F* = 1.26, *P* = 0.28).

Investigating correlated gene expressions in Experiment F, we found a positive association between α2M and proPO gene expression in the *Pasteuria* exposure treatment, which was, however, no longer significant after Bonferroni correction (Spearman Rank *R* = 0.77, *P* = 0.009, Bonferroni cut-off = 0.008). In the control treatment, no significant correlations were present.

## 3 Discussion

Understanding of the genetic factors that underlie host resistance to pathogens is an important first step towards elucidating coevolutionary dynamics. With this aim, the present study sought to gain insight into the inducible immune response of *D*. *magna*. Using sequence information from *D*. pulex, the first crustacean to have its genome sequenced; we used a candidate gene approach to assess the relevance of five putative immune effector genes in *D*. *magna* to one of its major pathogens, the bacterium *P*. *ramosa*. The host and pathogen samples used were well studied with respect to phenotypic responses to infection (e.g. Decaestecker et al., 2007; Carius et al., 2001), and the infectivity results obtained presently were in accordance with expectations. Specifically, for the German samples, Sp1 strain exposure yielded high infection levels for the GG4 *Daphnia* clone, but no infection for the GG13 clone; whereas exposure to the Sp8 strain yielded infections for GG13, but not for the GG4 *Daphnia* clone. The GG7 *Daphnia* clone was never infected and showed overall resistance to the two *Pasteuria* strains tested (this clone is nevertheless susceptible to other pathogen strains, see Carius et al., 2001). Exposure of the resurrected Belgian *Daphnia* clones to contemporary pathogens generated a high level of infection in both *Daphnia* clones, which is in agreement with the results of an earlier *Daphnia*–*Pasteuria* coevolution study (Decaestecker et al., 2007).

The tested candidate immune system genes – NOS1, NOS2, proPO, ARG and α2M – showed no significant modification of expression when the *Daphnia* were exposed to *Pasteuria*. And yet, we do expect an immune response by *Daphnia* in response to *Pasteuria* within the time-frames studied. Specifically, studies of cellular responses showed that infective combinations of host and pathogen genotypes generate large increases in the number of circulating haemocytes just a few hours after initial exposure (Auld et al., 2010). As the innate immune system is based primarily on phagocytic cells that generate reactive oxygen and nitrogen species, as well as other compounds (e.g. phenoloxidase) that can destroy or inhibit pathogens, we expected susceptible combinations of host and pathogen to show an increase in transcription of the chosen candidate genes. Earlier work on proPO expression in response to *Pasteuria* also provided reason for confidence in a candidate gene approach (Labbé and Little, 2009), but this proPO result could not be confirmed. The present study probably provides a more robust result because it doubled the number of control genes to which candidate immune system gene expressions could be referenced (Vandesompele et al., 2002; de Boer et al., 2009; Spanier et al., 2010), although intermittent and (subtle) condition-dependent upregulation of the proPO gene cannot be ruled out. Indeed, we cannot generally rule out the possibility that all our candidate genes show small changes in expression that our study lacked the power to detect, but we can conclude that none of them showed substantial changes. Moreover, our study investigated only expression (RNA) level activity, but responses to pathogen exposure may be more evident at the protein level, as can be revealed by proteomics studies (for *Daphnia* see Fröhlich et al., 2009; Schwerin et al., 2009).

Our study showed a tendency for a positive association between the proPO and α2M expression shortly after *Pasteuria* exposure. An interaction of this serpin in the proteolytic cascade of the PO-pathway has also been suggested by Wertheim et al. (2005), who found upregulation of genes coding for proteins with putative α2M domains when investigating genome-wide gene expression in *Drosophila* upon parasitoid attack. Additionally, Tang et al. (2008) showed that a protease inhibitor of the serpin family was involved in the regulation of the immune melanization process in *Drosophila*, however, in a negative way by inhibiting proteases that activate PO.

Our study also found evidence for clonal variation in constitutive NOS expression. In particular, in the Belgian population, there were clonal differences for the expression of NOS1, while in the German samples; NOS2 gene expression in the GG7 clone was consistently higher than it was in the other clones. There is as yet insufficient data to conclude if higher NOS constitutive expression may be linked to clonal differences in resistance or the level of virulence suffered during infection, but a previous study indicated that NOS could play a role in resistance to *Pasteuria* by supplementing the diet of *Daphnia* with amino acids that would either enhance or inhibit the production of NO (Labbé et al., 2009), and we suggest that further study of genetic variation in NO production may prove fruitful.

The molecular era has witnessed the discovery of deeply conserved pathways, including genes of the innate immune system (e.g. McTaggart et al., 2009; Cerenius et al., 2008). It is this conservation that offers the possibility of the candidate gene method, and it remains important to utilize this approach: although genomic and related technologies have advanced substantially in the past decade, it is not yet feasible to fully develop each system *de novo.* It is therefore necessary and potentially efficient to combine knowledge from genetic models (e.g. *Drosophila*) with models for the study of evolutionary ecology (i.e. *Daphnia*; Ebert, 2011; Colbourne et al., 2011). Nevertheless, testing expression of candidate immune system genes comes with risks and is prone to negative results. Specifically, despite the deep conservation of many biological pathways, including the ones studied presently, it is still likely that the inducible immune responses of genetic models (largely insects exposed to non-coevolved microbes or immune-stimulatory compounds) differs substantially from a crustacean exposed to its highly specialised bacterial pathogen. Indeed, the true diversity of immune responses is probably greatly under-appreciated given the limited taxonomic breadth of current models (Little et al., 2005).

## 4 Material and methods

### 4.1 *Daphnia*-pathogen system

We used three *D*. *magna* clones (GG4, GG7 and GG13) and two pathogen strains (Sp1 and Sp8) from a population originally collected near Gaarzerfeld in Germany (54°17′N 10°57′E), and which have been the focus of earlier studies on genetic specificity (Carius et al., 2001). We also studied two *Daphnia* clones (11.3 and 12.2) and one pathogen strain (Cl25.5_P2.1R3) originating from Belgium (50°51′N 04°43′E, OM2, Abdij van’t Park, Heverlee, see Decaestecker et al., 2007). The Belgian *Daphnia* clones and pathogen strains were derived from resting stages of layered pond sediments that can be reactivated after years or even decades (as performed in Decaestecker et al., 2007; Pauwels et al., 2007, 2010). The bacterial strains were isolated from the same depth of the sediment core as the *Daphnia* clones such that the *Daphnia* and the pathogens were isolated from the same time-frame (contemporary host and pathogen, see Decaestecker et al., 2007). Prior to the experiment, all *Daphnia* were maintained as clonal stock cultures in the laboratory, and the spores of the pathogen strains were kept frozen.

The pathogen used is the gram-positive bacterium *P*. *ramosa* that is an obligate, spore-forming endo-pathogen, infecting the hemolymph of *D*. *magna*. Infection causes severe fitness costs as the pathogen sterilizes the host shortly after infection (Ebert et al., 2004; Coors et al., 2008). Within the host, *Pasteuria* goes through a developmental process that culminates in the formation of spores. Host death is essential for transmission with the mature spores being released from the remains of dead infected hosts. *Pasteuria* spores are horizontally transmitted only, i.e. there is no evidence of transovarial infection (Ebert et al., 2004).

### 4.2 Selection of candidate immune system genes

We selected genes from a list of candidates previously identified in the completed *D*. *pulex* genome sequence by comparison to homologues from other arthropods (largely insects *Anopheles*, *Aedes*, *Drosophila*, *Tribolium*, and *Apis*, see McTaggart et al., 2009). The first gene we focused on was the *prophenoloxidase* (proPO) gene. The activation of the proPO cascade has been shown to be an important component of the humoral innate arthropod immune defense. Upon infection, the inactive zymogen proPO is converted into phenoloxidase (PO), which catalyzes the oxidation of phenols to quinones that then polymerize into melanin, resulting in melanization of the pathogen. PO-activity is also associated with the production of reactive oxygen species (ROS) enhancing pathogen destruction and expression of proPO has been shown to be associated with immune challenge in several species (Labbé and Little, 2009; Wertheim et al., 2005; Tang et al., 2008; Cerenius et al., 2008).

A second investigated candidate gene is the *alpha-2-macroglobulin* (α2M) gene, which is a thio-ester containing protein (TEP gene) coding for a serpin (serin protease inhibitor) that has been shown to inhibit extracellular pathogen serin proteases and gram-positive bacterial sepsis in vertebrates (deBoer et al., 1993). The α2M gene is potentially involved in resistance against *Pasteuria*, as this is a gram-positive extracellular bacterial pathogen of the *Daphnia* hemolymph (Ebert, 2005). Moreover, Little et al. (2004) showed that the *Daphnia* gene sequence of the bait region of α2M is under positive selection, which suggests that it may be involved in an ‘arms race’ with pathogens.

The three last candidate genes investigated were two *Nitric Oxide Synthase* genes (NOS1, NOS2) and an *arginase* gene. NOS genes encode the *Nitric Oxide Synthase* that, by converting l-arginine into l-citrulline, produces a highly reactive free radical gas, the nitric oxide (NO) with many biological functions, including defense against pathogens (see review in Tripathi et al., 2007). NO reacts with oxygen to create many oxygen-species toxic to many pathogens (Tripathi et al., 2007; Rivero, 2006). The immunity role of NO has been demonstrated in a range of organisms, notably through up-regulation of NOS expression following infection (e.g. Dimopoulos et al., 1998; Foley and O’Farrell, 2003; Yeh et al., 2006). Contrary to many species, two copies of the gene are present in *Daphnia* (Labbé et al., 2009). NOS and *arginase* compete over the same substrate, the arginine, and therefore activation of *arginase* may be a mechanism employed by pathogens to limit host production of NO, thus increasing host susceptibility (Vincendeau et al., 2003).

### 4.3 Exposure protocols

Before exposing hosts to pathogen spores, host maternal lines were raised in standard conditions for at least three generations to control for maternal effects. From each German clone, twelve independent replicates with four female *Daphnia* were maintained in jars containing 60 mL of artificial medium (Klüttgen et al., 1994) and were fed 3.5 × 10^6^ chemostat grown *Chlorella* sp. algal cells per *Daphnia* per day. The experimental generation was created by mixing all the offspring collected in the twelve jars and randomly putting four individuals per experimental jar. In the first two generations, each Belgian clone was kept in three replicates with four individuals in 60 mL jars with 3.5 × 10^6^ algal cells per *Daphnia* per day. In the last maternal generation, each replicate of a Belgian clone was kept in 2 L jars with 10.5 × 10^6^ algal cells per *Daphnia* per day. All *Daphnia* were maintained in experimental jars, randomized in temperature-controlled (20 °C) incubators with a light:dark cycle of 12:12 h. Medium was changed every four to five days.

Four independent short-term experiments (1–48 h) were performed comparing expression of several candidate genes at several time-points after exposure. We used the same protocol as described in detail in Labbé and Little (2009). Briefly, we exposed replicates of four to five day-old *Daphnia* to a solution of *Pasteuria* spores (strains Sp1 (Exp. A, B, and D) or Sp8 (Exp. C), same strains as in Carius et al., 2001) or to a placebo solution in 1.5 mL eppendorf tubes. After an exposure of two hours, the *Daphnia* were placed in a 100 mL jar and reared under standardized conditions (see above). Three replicate jars for each exposure treatment were randomly collected 1, 2, 6, 12, 24 and 48 h after the end of the exposure. Additionally, three jars were also collected at time 0, prior to exposure, to be used as start reference and negative control (in total 21 experimental jars per exposure treatment were set up at the beginning of the experiment). For each time-point, the four *Daphnia* of a same jar were transferred to one eppendorf tube with 200 μL of RNAlater™ (Ambion) and stored at −20 °C for later extraction: we thus had three replicates per time-point and exposure treatment, consisting in a mix of four *Daphnia*. qRT-PCR was then performed on this mix of four *Daphnia* for each harvested jar. Several replicates of each treatment were kept in rearing conditions until day sixteen, in order to estimate infection success.

In all four short-term experiments (A–D), we exposed individuals from the *Daphnia* clones to a *Pasteuria* spore or placebo solution. We then analysed the expression of different *Daphnia* candidate immune system genes (proPO, NOS1, NOS2, *arginase* and α2M) for each replicate of the different treatments and time-points, relative to two reference genes (*actin* and GAPDH). Further information on the description of the *Daphnia* clones and *Pasteuria* strains used and of the genes analysed in each experiment is in Table 1. Experiment A has been described in previous studies (see Labbé and Little, 2009; Labbé et al., 2009), but presently we used the same individuals (frozen cDNA) for additional analyses for two more genes: *arginase* and the reference gene GAPDH.

In the long-term experiment (Exp. E), we used *Daphnia* clones (GG4 and GG7) and exposed them to a pathogen (Sp1 strain) or a placebo solution for 48 h. The protocol was largely similar to the one used for the short-term experiments, described above, but differed in several respects. Here we used six individuals, reared in 200 mL jars and exposed them to a pathogen or a placebo solution in 60 mL jars with a low quantity of food (3.5 × 10^6^ chemostat grown *Chlorella* sp. algal cells per *Daphnia* per day). We also added sterile sand, which was stirred twice during the 48 h of the exposure treatment. The addition of sand and the low food both increased bottom grazing behaviour, which increases the chances of *Daphnia* encountering the pathogen spores and reduces variation among individual exposures (Decaestecker et al., 2002). Samples were collected after 12 and 24 h, and then 2, 4, 8 and 16 days after the start of the exposure treatment. Six replicates of each time-point were collected for each treatment, plus six replicates collected at time 0, just before the start of the exposure treatment (used as negative control). On day sixteen, samples were investigated for signs of infection. For each of the six replicates of the different treatments and time-points, we analysed the expression of two reference genes *actin* and GAPDH, and that of four candidate immune system genes, proPO, NOS1, NOS2 and *arginase*.

In the short-term experiments, some trends were seen around two hours after exposure for some of the candidate genes tested (see Section 2 and Labbé and Little, 2009; Labbé et al., 2009). To verify these trends, we performed a further short-term experiment that focused on the two-hours time point, but we studied more replicates (*N* = 10; Exp. F). We used exactly the same protocol as for short-term Experiment A&B with *Daphnia* clone GG4 exposed to pathogen Sp1 strain or a placebo solution for two hours. For each replicate of the different treatments, we analysed the expression of the two reference genes (*actin* and GAPDH) and that of the candidate genes (proPO, NOS1 and α2M). Given that the exposure of this clone earlier resulted in significant exposure effects for some of the candidate genes tested, we also tested for correlated expression (Spearman Rank Correlation) of the different genes, separately for the pathogen exposure and control treatment.

### 4.4 Expression analysis

RNA was extracted using the RNAeasy midi Kit (Qiagen), according to manufacturer's instructions. The RNA was further purified with RNAse-Free DNAse (Promega). Two microliters RNA was reverse-transcribed into cDNA using the Promega Reverse Transcription System kit according to manufacturer instructions. cDNA was diluted five fold by adding 80 μL of H_2_O to each tube.

A ∼100 bp fragment of *actin* and a ∼100 bp fragment of GAPDH were PCR amplified separately using primers from Heckmann et al. (2006), a 101 bp fragment of proPO was amplified separately using primers from Labbé and Little (2009), a 99 bp fragment of NOS1 and a 106 bp fragment of NOS2 were amplified separately using specific primers from Labbé et al. (2009). For *arginase* and α2M, specific primers were designed from published sequences using the online software Primer3 (http://www.bioinformatics.nl/cgi-bin/primer3plus/primer3plus.cgi). For *arginase*, primers were designed using *D*. *melanogaster* sequence as a reference (Samson, 2000) blasted on the *D*. *magna* NCBI EST library (Watanabe et al., 2005). By blasting, we identified two sequences (BJ925714 and BJ925715) which were used to design a pair of primers amplifying a 106 bp fragment of *D. magna arginase*: ArgQF1 5′ TGGTCTCCGGGATGTAGAAC 3′/ArgQR1 3′ GACGGCTTCTTTGATGCCTA 5′. For α2M, primers were designed using a 300 bp fragment extracted from seven *D. magna* sequences as a reference (Little et al., 2004, NCBI Nucleotide library AY540086 to AY540092). This fragment was used to design a pair of primers amplifying a 96 bp fragment of a *D. magna* sequence α2MQF2 5′ TTTTTAGTGCGACGGAAGATGTG 3′/α2MQR2 3′ AAAGCCAGGTCTCGGGAAAGTAG 5′).

Relative qRT-PCR was performed using the Roche LightCycler^®^ 480. We added cDNA (1 μL for *actin*, GAPDH, proPO, α2M or *arginase*, and 2 μL for NOS1 and NOS2) and 0.5 μL of each primer to 8 μL of SYBR Green I Master mix (Roche). Cycling conditions were 95 °C, 5 min followed by 45 cycles of 95 °C for 10 s, 58 °C for 10 s and 72 °C for 10 s. Quantification of the different genes relative to *actin* or GAPDH was performed using the Roche LightCycler^®^ 480 software, using the maximum secondary derivative method. Following constructor recommendations, quantifications were considered valid only if efficiency was very close to 2. As a second check-point, we used sequential 4-folds dilutions of a known sample to build standard curves (5 points). Quantifications were considered only when the slope was close to 3, the expected value if dilutions are good. If a sample failed these requirements, it was re-analysed.

### 4.5 Statistical analysis

For each gene and for each replicate (genotype, time-point and treatment), we obtained expression data; one set relative to *actin* and one set relative to GAPDH. As the efficiencies were similar and to combine the information from the two reference genes (i.e. to minimise the risk of being driven to false variations by one of the references, as recommended in Heckmann et al. (2006), we normalised the data, as the raw quantitative expressions of the two reference genes differed. To do that, we divided each *actin*-related data point by the mean of all *actin*-related data (so that the mean of the normalised *actin*-related data equals 1); we did the same for the GAPDH-related data (so that the mean of the normalised GAPDH-related data also equals 1). We then took the mean of the two values for each replicate of each time-point and treatment as the synthetic expression data.

We first investigated genotype, exposure and time point effects on the expression data. Experiments A and B are replicates of the same protocol, so we analysed them in a single statistical model (i.e. they are analysed together but not pooled). We used the two levels categorical variable Expe (Experiment) to refer to each experiment (A or B) and test whether the gene expressions in these experiments were different or not. The synthetic data were fitted to the generalized linear model (GLM): Log(Expression) = Geno × Expo × Time × Expe. Geno refers to the *Daphnia* genotype, a categorical variable with three levels (GG4, GG7 and GG13). Expo (Exposure) is a categorical variable with two levels that refers to the exposed versus not-exposed (placebo) pathogen treatment. Time is a continuous variable (referring to the hours after pathogen exposure). Log-transformation of the response variable ensured the normal distribution of residuals. For Experiments C, D and E, the synthetic data were independently fitted to the GLM: Log(Expression) = Geno × Expo × Time.

In all cases, the initial model was simplified starting from highest-level interaction down to the main effects, according to Crawley (2007). Models were compared using *F*-tests. Normality of the data was tested using the Shapiro–Wilk normality test.

We were interested in the three-way interaction Geno × Expo × Time, which would indicate an increase/decrease in expression after pathogen exposure between the *Daphnia* clones during the experiment. The other terms of interest were the Geno × Expo interaction which would indicate that *Daphnia* clones differed in their reaction to pathogen exposure, and the Time × Expo interaction which would indicate a variable reaction to pathogen exposure through time, but similar between clones. Finally, a significant Geno or Expo main effect would, respectively, indicate a basal expression that differed between the *Daphnia* clones or a constant reaction to exposure between the different *Daphnia* clones tested.

To prevent multiple testing errors, a sequential Bonferroni procedure (Hochberg, 1988) was applied to the tests of the different genes in a particular experiment.

Analysis was performed using the R package (http://www.r-project.org/) and Carpenter et al. (2009).

## Figures and Tables

**Table 1:** Set-up experiments and results on average infection rates. For each experiment (Exp.), the *Daphnia* clones and parasite strains used and the genes investigated are indicated. Some details of the protocols are also given: parasite doses are given in number of spores per *Daphnia*, time is given in hours (h) or days (d) and infection rates (0 = no infection, 1 = all *Daphnia* infected) are given for each clone (conserved order).

Exp.	Figure	*Daphnia* clone	Parasite	Dose	Exposure time	Time points	Genes investigated	Infection rate
A	Fig. 1	GG4/GG7	Sp1	100,000	2 h	0 h, 1 h, 2 h, 6 h, 12 h, 24 h, 48 h	*actin*, GAPDH, *arginase*, proPO, NOS1, NOS2	0.73/0
B	Fig. 1	GG4/GG7/GG13	Sp1	100,000	2 h	0 h, 1 h, 2 h, 6 h, 12 h, 24 h, 48 h	*actin*, GAPDH, *arginase*, proPO, NOS1, NOS2	1/0/0
C	Fig. 2	GG4/GG7/GG13	Sp8	150,000	2 h	0 h, 1 h, 2 h, 4 h, 6 h, 12 h, 24 h, 48 h	*actin*, GAPDH, *arginase*, proPO, NOS1, NOS2	0/0/0.27
D	Fig. 3	11.3/12.2	Cl25.5_P2.1R3	100,000	2 h	0 h, 1 h, 2 h, 6 h, 12 h, 24 h, 48 h	*actin*, GAPDH, proPO, NOS1, α2M	0.91/0.61
E	Fig. 4	GG4/GG7	Sp1	20,000	48 h	12 h, 24 h, 2d, 6d, 8d, 16d	*actin*, GAPDH, *arginase*, proPO, NOS1, NOS2	1/0
F	Fig. 5	GG4	Sp1	100,000	2 h	2 h	*actin*, GAPDH, proPO, NOS1, α2M	1

**Table 2:** Results of GLM on candidate gene expression in the exposed versus non-exposed treatment in Experiments A, B, C and E. Only the terms significant for at least one gene are presented (all other terms showed a *P*-value *P* > 0.05). Geno refers to the *Daphnia* genotype, Expo to the exposed versus not-exposed treatment, Time is the time since exposure (see Section 4). When the Geno main effect was significant, closest genotypes were grouped and the grouping effect was tested (GG4 = GG13). Significant terms are bolded. Experiments A and B were replicates of the same protocol, thus they were analysed using the same statistical model, with the variable Expe (see Section 4).

Exp.	Test	proPO		NOS1		NOS2		*Arginase*	
		*F*	*P*	*F*	*P*	*F*	*P*	*F*	*P*
A&B	Geno:Time	1.05	0.352	**3.58**	**0.030**	0.79	0.457	1.91	0.151
	Geno:Expe	0.13	0.721	**4.36**	**0.038**	3.31	0.071	0.09	0.767
	Expo:Expe	0.01	0.914	0.39	0.532	0.12	0.724	**8.49**	**0.004**
	Time:Expe	0.06	0.804	**4.55**	**0.034**	0.08	0.776	0.00	0.994
	Geno	1.37	0.258	–	–	**19.0**	**0.000**	0.22	0.805
	GG4 = GG13	–	–	–	–	0.03	0.863	–	–
	Expe	2.58	0.110	–	–	**7.40**	**0.007**	–	–
	Time	**17.38**	**0.000**	–	–	**6.62**	**0.011**	3.06	0.082

C	Expo	**5.99**	**0.016**	0.43	0.512	0.21	0.649	1.13	0.290
	Geno	0.01	0.995	3.76	0.055	**21.1**	**0.000**	0.14	0.711
	GG4 = GG13	–	–	–	–	1.09	0.298	–	–
	Time	**4.53**	**0.035**	2.44	0.091	1.00	0.318	0.29	0.747

E	Expo	**5.37**	**0.022**	0.11	0.737	0.47	0.492	1.18	0.279
	Geno	3.59	0.060	1.78	0.184	**34.2**	**0.000**	0.69	0.407
	Time	**194.3**	**0.000**	**50.5**	**0.000**	3.72	0.056	**15.2**	**0.000**

**Table 3:** Results of GLM on candidate gene expression in the exposed versus non-exposed treatment in Experiment D. Only the terms significant for at least one gene are presented (all other terms showed a *P*-value *P* > 0.05). Geno refers to the *Daphnia* genotype, Time is the time since exposure (see Section 4). Significant terms are bolded, those close to significance are in italics.

Test	proPO		NOS1		α2M	
	*F*	*P*	*F*	*P*	*F*	*P*
Geno:Time	0.63	0.431	0.00	0.952	3.70	*0.058*
Geno	3.40	0.069	**27.97**	**0.000**	0.40	0.527
Time	**10.07**	**0.002**	2.46	0.121	**56.38**	**0.000**
